# Simulations and Measurements of Human Middle Ear Vibrations Using Multi-Body Systems and Laser-Doppler Vibrometry with the Floating Mass Transducer

**DOI:** 10.3390/ma6104675

**Published:** 2013-10-22

**Authors:** Frank Böhnke, Theodor Bretan, Stefan Lehner, Tobias Strenger

**Affiliations:** 1 Department of Otorhinolaryngology, Head and Neck Surgery, Klinikum rechts der Isar, Technical University Munich, Ismaninger Str. 22, Munich 81675, Germany; E-Mail: t.strenger@lrz.tum.de; 2 Department of Applied Sciences and Mechatronics, Munich University of Applied Sciences, Loth Str. 34, Munich 80335, Germany; E-Mails: bretan@hm.edu (T.B.); lehner@lfe.mw.tum.de (S.L.)

**Keywords:** middle ear, μCT, Multi-body System (MBS), floating mass transducer (FMT), laser-Doppler vibrometry (LDV)

## Abstract

The transfer characteristic of the human middle ear with an applied middle ear implant (floating mass transducer) is examined computationally with a Multi-body System approach and compared with experimental results. For this purpose, the geometry of the middle ear was reconstructed from μ-computer tomography slice data and prepared for a Multi-body System simulation. The transfer function of the floating mass transducer, which is the ratio of the input voltage and the generated force, is derived based on a physical context. The numerical results obtained with the Multi-body System approach are compared with experimental results by Laser Doppler measurements of the stapes footplate velocities of five different specimens. Although slightly differing anatomical structures were used for the calculation and the measurement, a high correspondence with respect to the course of stapes footplate displacement along the frequency was found. Notably, a notch at frequencies just below 1 kHz occurred. Additionally, phase courses of stapes footplate displacements were determined computationally if possible and compared with experimental results. The examinations were undertaken to quantify stapes footplate displacements in the clinical practice of middle ear implants and, also, to develop fitting strategies on a physical basis for hearing impaired patients aided with middle ear implants.

## 1. Introduction

The human middle ear (ME) is a mechanical structure that acts as a transducer to adapt the high acoustical impedance of the lymph of the inner ear to the low impedance of the surrounding air. This impedance ratio of water:air (≈3500) leads to a nearly complete reflection of acoustical waves without the middle ear and, thus, would impede acoustic communication with high sensitivity.

Early models of the middle ear represented the complex biomechanical system by electrical network components with inductance representing mass, resistors representing damping and capacitors representing stiffness [1]. Anatomical structures, like ligaments and bones, are incorporated by complex impedances. In order to map the real dynamic mechanical behavior of the middle ear, an electromechanical analogy was used. This approach is efficient, due to the simplification of a multi-degree of freedom system to a system with a strongly reduced number of variables, which could be evaluated by the limited computing power at that time. However, with this approach, it was difficult to examine the mechanical impact of the deficiency of ligaments or muscles on the displacement of the stapes footplate. With the evolution of numerical computation techniques and finite element approaches, more complex simulations could be implemented. Due to the high computational load, the ligaments could not be considered completely [2].

The geometrical reconstruction of the auditory ossicles based on micro-computer-tomography (μCT) imaging [3] and orthogonal-plane fluorescence optical-sectioning microscopy (OPFOS) [4] is possible with a subsequent transfer of data for further numerical evaluation. Here, we use a reconstruction based on μCT slice data. Even though the inclusion of implantable middle ear hearing aids was successful with a finite element (FEM) approach [5], few numerical results of, e.g., stapes footplate displacements are presented up to now. An alternative approach to simulate the mechanical behavior of the middle ear is the technique of Multi-body Systems (MBSs) [6]. In this case, a mechanical object is represented by the center of gravity and the mass moment of inertia. Because of this reduction of complex geometrical structures to a few parameters, a considerable savings of computational work is achieved in the case of MBS. Another benefit of the MBS representation in comparison to FEM is the significant simplification in coupling structures with different material properties. In the case of FEM, contact and interface areas must be defined and handled, whereas with MBS, lumped joints implemented by the SIMPACK (Simulation of Multi-body System Package, SIMPACK AG, Gilching, Germany) software [7] are sufficient to realize the coupling of structures easily. Of course, the MBS approach is not applicable for simulations with fluid-solid or gas-solid interfaces initially, but this was not aimed at in this study. Another important advantage of FEM simulations is their ability to represent the complex vibration patterns of elastic structures. In the case of the middle ear, this could be the tympanic membrane, which was already modeled in 1978 by Funnel and Laszlo for cat ears using FEM [8]. Especially in modeling cochlear mechanics, with its complex vibrations of the spatially extended basilar membrane, FEM is superior to MBS. However, in the case of the middle ear, the chain of discrete rigid bodies (the ossicles) is represented by MBS sufficiently. Here, the MBS method is implemented to simulate the dynamics of middle ear vibrations, including the influence of a middle ear implant, the floating mass transducer (FMT).

## 2. Methods

### 2.1. Reconstruction of the Middle Ear

**Figure 1:**
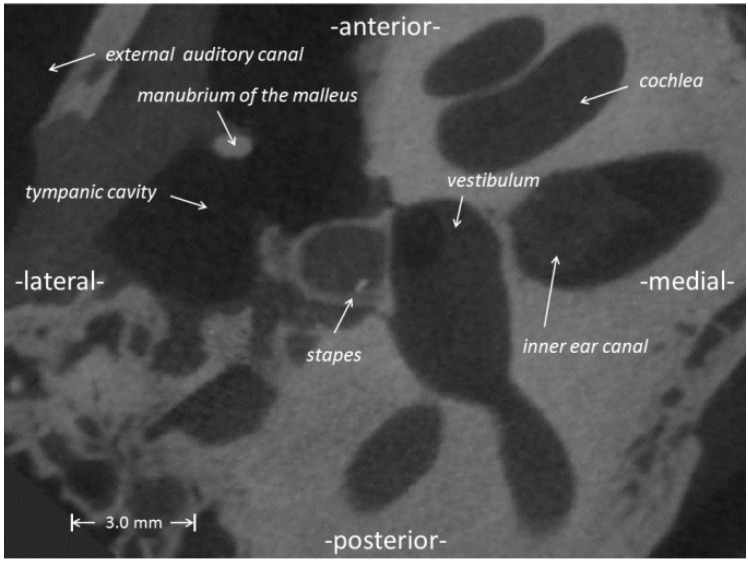
Micro-computer-tomography (μCT) slice image showing a part of a human temporal bone with cross-sections of the stapes and the manubrium of the malleus. The light gray areas depict bone. Orientations are marked at the image borders.

A Multi-body System (MBS) of a left middle ear is based on slice data obtained from a human temporal bone with a μCT (Imtec^®^, Ardmore, PA, USA) with an analysis resolution of 50 μm in space and manual segmentation using level detection reconstruction of the visualization software, AMIRA^®^ (Visualization Sciences Group). The frozen specimen was thawed and conserved in formaldehyde. Indeed, the impregnation with formaldehyde had an influence on the size of ligaments, which will increase by swelling. Though all six ligaments of the middle ear were reconstructed, their geometries are not used in this study, and they are represented by Voigt elements (spring/dashpot) of the MBS. One slice presenting a cross-section of the stapes is shown in Figure 1 exemplarily. The dark gray area below (right) the stapes footplate is the space filled with perilymph *in vivo*. The light gray areas represent zones of compact bone.

**Figure 2:**
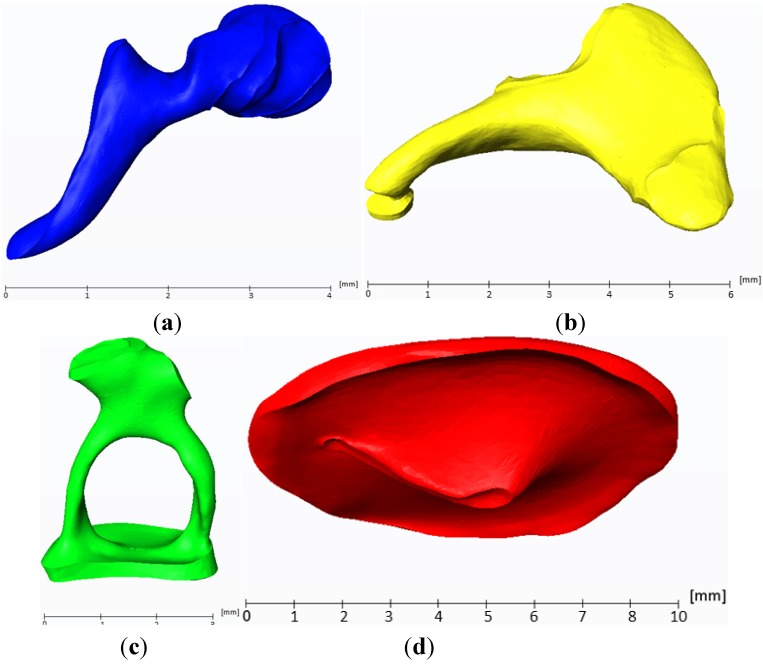
(**a**) 3D reconstruction of the malleus obtained from μCT slice data. On the left, the curved shape of the manubrium of the malleus is seen. On the right, the emargination of the incudomalleolar joint in the malleus head is seen; (**b**) 3D reconstruction of the incus obtained from μCT slice data; (**c**) 3D reconstruction of the stapes obtained from μCT slice data; (**d**) 3D reconstruction of the tympanic membrane (TM) obtained from μCT slice data.

The malleus, incus and stapes are shown in Figure 2a–d. All six ligaments and both muscles (musculus stapedius and musculus tensor tympani) of the middle ear were segmented and reconstructed additionally, but are not shown, since they are not represented as individual multi-body elements in this MBS simulation.

The reconstructed stapes (Figure 2c) has a maximum longitudinal dimension of 3 mm and an area of 3.8 mm^2^. A current study [9] of 53 human stapes states a length of the footplate with an average of 2.81 mm (±0.158 mm standard deviation), a width of 1.27 mm (±0.109 mm standard deviation) and an area of 3.03 mm^2^ (±0.331 mm^2^ standard deviation). The tympanic membrane (Figure 2d) has a maximum longitudinal dimension of 10 mm and an area of 70 mm^2^. Because the tympanic membrane is thin (80–100 μm [10]), special care had to be taken for an appropriate reconstruction. For this purpose, the μCT dataset was supplied with additional coordinate systems, whose individual axes were positioned in the plane of the TM. With this method, the borders of the TM could be seen exactly. A biased thickness due to an angulated slice could therefore be prevented. The outer border of the tympanic membrane is framed by the annulus fibrocartilagineus and is included in Figure 2d. The significant curvature and the area, which couples the tympanic membrane to the malleus, can be seen.

### 2.2. Multi-Body Systems

**Figure 3:**
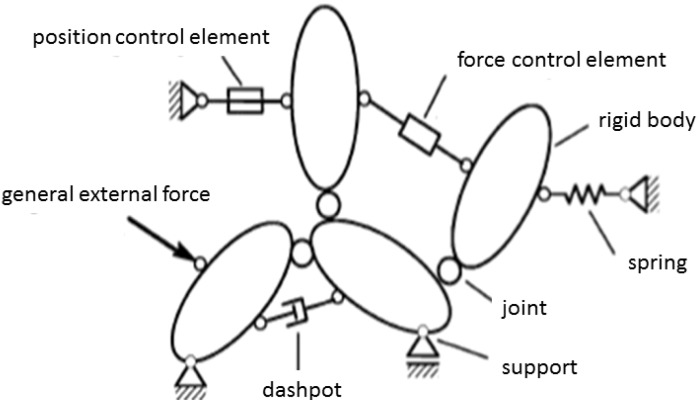
General Multi-body System (MBS) with components (Reprinted with permission from [10]. Copyright 2013 Springer).

Initially, a general description of the Multi-body Systems (MBSs) method is described. A MBS consists of one or more rigid bodies, which are connected in between and to the environment by massless joints, springs, force elements and others, as shown in Figure 3 [11].

For this work, the software package, SIMPACK is used. This MBS software uses *bodies, markers, sensors, joints* and *force elements.* For the evaluation of movements, SIMPACK defines a constant inertial frame. Each rigid *body* has a constant reference system in relation to the inertial system. *Markers* are defined in the gravity center of each *body* and are characterized by the position and orientation in relation to the reference system. *Sensors* are used to calculate kinematic data (position, velocity and acceleration) between *markers*. *Force elements* are connections between *bodies* or between a *body* and its environment, which might apply forces and moments to the *body*.

The number of degrees of freedom (DOFs) of an MBS is determined by the equation of Grübler:

(1) DOF=6 ·(n−j−1)+∑N

where *n* is the number of *bodies*, including the inertial system, *j* is the number of *joints* (hinges), and *N* is the number of the DOFs of the *joints*. In the case of the middle ear as a mechanical system, the small displacements around the resting position allow a treatment of its mechanical behavior by the linearized equations of motion [12]:

(2) $$M\cdot \ddot{x}(t)+\;D\cdot \dot{x}(t)+K\cdot x(t)=\;F(t)$$

***M***, mass matrix; ***D***, damping matrix; ***K***, stiffness matrix; ***F***(*t*), vector of forces at time *t*; x(t), vector of displacements at time *t*, $\dot{x}(t)$ vector of velocities at time t; $\ddot{x}(t)$, vector of accelerations at time *t*.

The initial state of the system is determined by the equation for the static equilibrium:

(3) $$K\cdot x(t)=\;F_{\text{Stat}}$$

$\;F_{\text{Stat}}\;$, vector of static forces.

### 2.3. Multi-Body System (MBS) of the Human Middle Ear 

**Figure 4:**
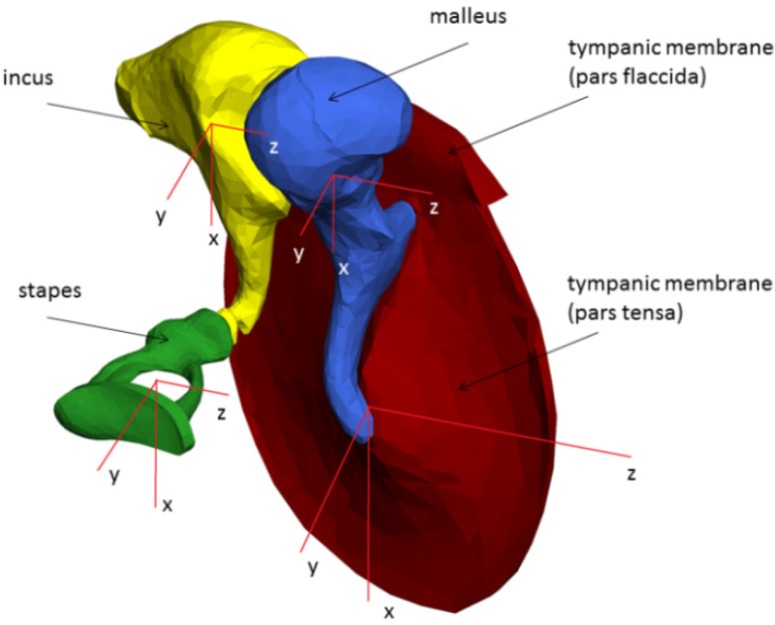
Tympanic membrane (red), malleus (blue), incus (yellow) and stapes (green), including local coordinate systems originating in the centers of gravity, are given for each rigid body.

The MBS of the human middle ear is based on a μCT dataset, reducing the effective data size to be processed by a factor of 170. Each body (pars tensa, pars flaccida, malleus, incus, stapes and FMT) is assigned a user-defined coordinate system, whose origin is identical to its center of gravity (Figure 4).

The alignment of the individual coordinate system for each body is identical to the alignment of the global coordinate system. Table 1 presents densities, masses, volumes and areas of all rigid bodies. The densities are taken from the literature and were assumed as homogeneously distributed inside the bodies. All calculated masses, volumes and areas of Table 1 are the results of the software, Solid Edge ST2, using the reconstructed .stl files from the visualization software, AMIRA^®^.

**Figure 5:**
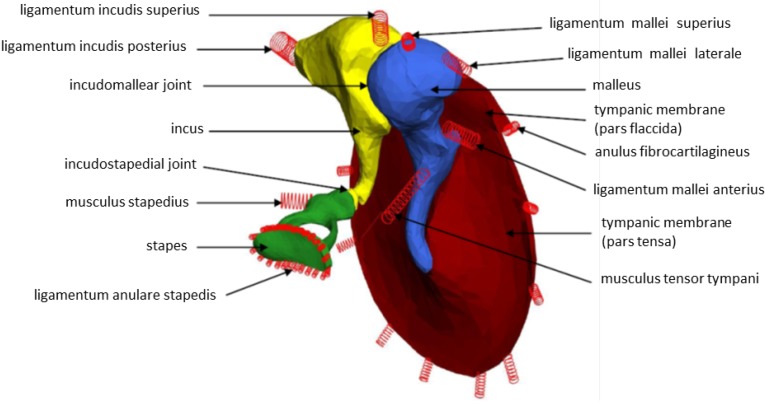
MBS middle ear model, including springs representing ligaments and muscles.

The viscoelastic properties of the six ligaments and the two muscles of the middle ear were modeled as massless Voigt force elements. A Voigt force element is a spring and a dashpot in parallel. Figure 5 shows the rigid bodies with additional Voigt force elements diagrammed as springs. For a condensed description, only the joint between the incus and the stapes (incudostapedial joint) is described exemplarily in the following.

**Table 1:** Parameters of the rigid bodies constituting the MBS of the middle ear model and the floating mass transducer (FMT).

Rigid body	Density (kg/m^3^)	Calculated mass (mg)	Calculated volume (mm^3^)	Calculated area (mm^2^)
Pars tensa	1200 (Koike and Wada)	10.01	8.34	131.21
Pars flaccida	1200 (Koike and Wada)	1.4	1.17	15.37
Malleus	3590 (Lee)	57.21	15.94	44.87
Incus	3230 (Lee)	46.31	14.34	42.14
Stapes	2200 (Lee)	3.94	1.79	16.18
FMT Samarium	7520	31.68	40.2	19.95

Between the stapes and the incus, a small slice of cartilage (thickness 0.2 mm) is positioned. The incudostapedial joint is modeled as a joint with six degrees of freedom. The viscoelastic properties of the synovial joint between incus and stapes are represented by two unilateral contact force elements and one rotation force element. The spring constant of the first unilateral contact force element are 2000 N/m for the *y*- and *z*-axis and 3000 N/m for the *x*-axis [13]. The damping constant is 5 × 10^−4^ Ns/m along the *y*- and *z*-axis and 5 × 10^−3^ Ns/m along the *x*-axis [12]. The second unilateral contact force element has a spring constant −2000 N/m [11] and a damping constant of 5 × 10^−4^ Ns/m [11], both along the z-axis. The rotational movements were represented by rotational force elements for all three axes. The spring constant was 0.1 Nm/rad (*x*-axis), 0.004 Nm/rad (*y*-axis) and 0.001 Nm/rad (*z*-axis), and the damping was 0 N·m·s/rad in all cases [12].

Though the tympanic membrane (Figure 2d) exhibits a complex spatial vibration pattern, which is usually modeled by the finite element method, it was sufficient to capture it also as one rigid body. The reason for that is the main purpose of this work, namely, to quantify the stapes footplate displacement with an FMT applied at the manubrium of the incus.

The tympanum annulus fibrocartilagineus is modeled by 12 spring elements, each having a spring constant of 212 N/m and 10^−2^ Ns/m damping constants for all axes.

The ligamentum anulare stapedius is idealized by 25 force elements (spring-damper), each having a stiffness of 9 N/m and a damping constant of 0.02 Ns/m. To consider the fluid load of the lymph (cochlear impedance), one additional spring with a stiffness of 200 N/m was placed at the stapes footplate [6,14]. Subsequent to the complete implementation of the MBS middle ear model and achievement of the static equilibrium, SIMPACK creates and solves the equations of motion (2).

### 2.4. Laser-Doppler Vibrometry (LDV)

The velocity of the stapes footplate was measured using a single point fiber laser-Doppler vibrometer and its associated vibrometer controller (OFV-512 and OFV-5000, Polytec, Waldbronn, Germany). The laser beam was reflected by a reflective tape placed on the stapes footplate. The mass of the reflective tape can be neglected. Five fresh frozen human temporal bones were examined and thawed immediately before the experiments. The Doppler-shifted signal was decoded to produce a signal proportional to the stapes velocity. To compare the measured velocities with the calculated displacements, the sinusoidal velocities were divided by a factor *i* × ω for each frequency for the purpose of integration. 

### 2.5. Floating Mass Transducer (FMT)

**Figure 6:**
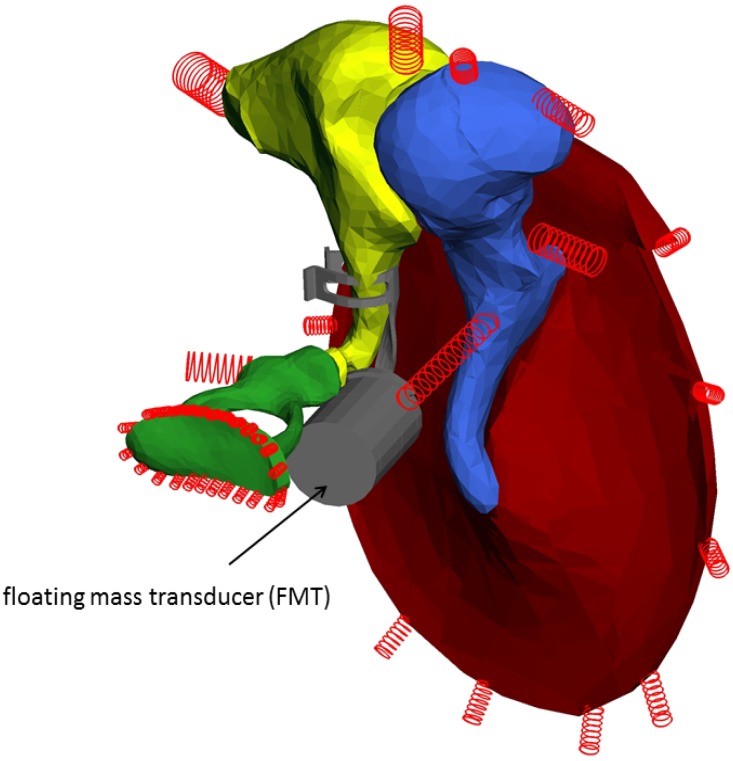
Middle ear with FMT (gray) crimped at the long process of the incus (yellow).

**Figure 7:**
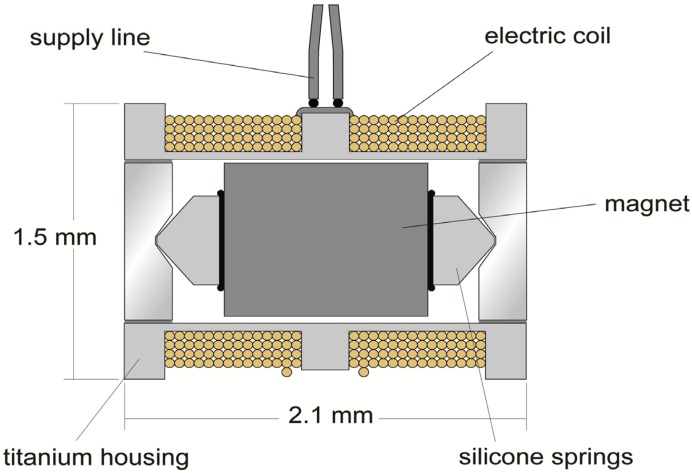
Cross-section of the floating mass transducer (Reprinted from [15]).

The FMT is an electromagnetic device, which is crimped usually at the long process of the incus and transfers acoustic energy to the hearing impaired ear (Figure 6). Figure 7 [15,16] depicts a cross-section of the device. The magnetically-polarized samarium core with its permanent magnetization is in the center of the element. The elastic silicone elements (springs) to the left and right of the magnet support the magnet and reduce the distortions, which are produced by the transient reversal of magnet displacement relative to the housing. The housing is wrapped by a gold wire, whose cross-sections are represented as small circles in the figure.

The exact force generation of the FMT considering the moving magnet inside the housing and its displacement reversal according to the elastic springs relative to the housing is difficult to calculate. Therefore, a simplified approach for the estimation of force production considering only a unidirectional treatment is presented. The result has a correct order of magnitude compared to experiments [17].

According to Lorentz, the force, *F*, acting on the permanent magnet inside the FMT is proportional to the electric current, *I*, flowing through the electric coil (gold wire) multiplied by the effective length of the coil and the remnant magnetic induction *B_r_* = 1 T (Tesla) in the case of samarium-cobalt.

(4) $$F=\frac{I\;\times \;l\;\times \;B_{r}}{\mu_{0}}$$

The length, *l*, of the wound gold wire with an average radius *R =* 0.75 mm and *n =* 220 turns is 1.036 m*.* Using the magnetic field constant μ_0_ = 4 × π × 10^−7^ Vs/Am and considering the equivalence of 1 N and 10^7^ A^2^ arising from the conversion of electromagnetic cgs units 1 Coulomb ≙ 10^−1^ cm^0.5^·g ^0.5^ with an electric current *I =* 20 mA (1 V/50 Ω)*,* the axial force is:

(5) $$F=\;1.648\;\times \;10^{-3}\left[\;N\;\right]$$

which is near the force value given by the FMT transmission factor of 3 × 10 ^−3^ N/V determined experimentally by Strenger [17]. To determine this force, the FMT was positioned on a spring, and the velocity of the current driven system was measured by an LDV. Because the mass m1 of the magnet of the FMT is nearly decoupled from the mass m2 of the titanium housing and the attached spring, two resonance peaks of the measured velocity occur along the frequency. Adapting this double resonance behavior to two differential equations of type (2) permits the identification of the absolute value of the force, *F*, which is equal, but has the opposite sign by parameter fitting of the two differential equations [17].

## 3. Results

According to the MBS model of the human middle ear, the stapes footplate vector with magnitudes of displacements (SFD) and phases of displacements are (SFP) were evaluated for stimulation frequencies between 100 Hz and 10 kHz.

### 3.1. Calculated and Measured Stapes Footplate Displacements (SFD) 

**Figure 8:**
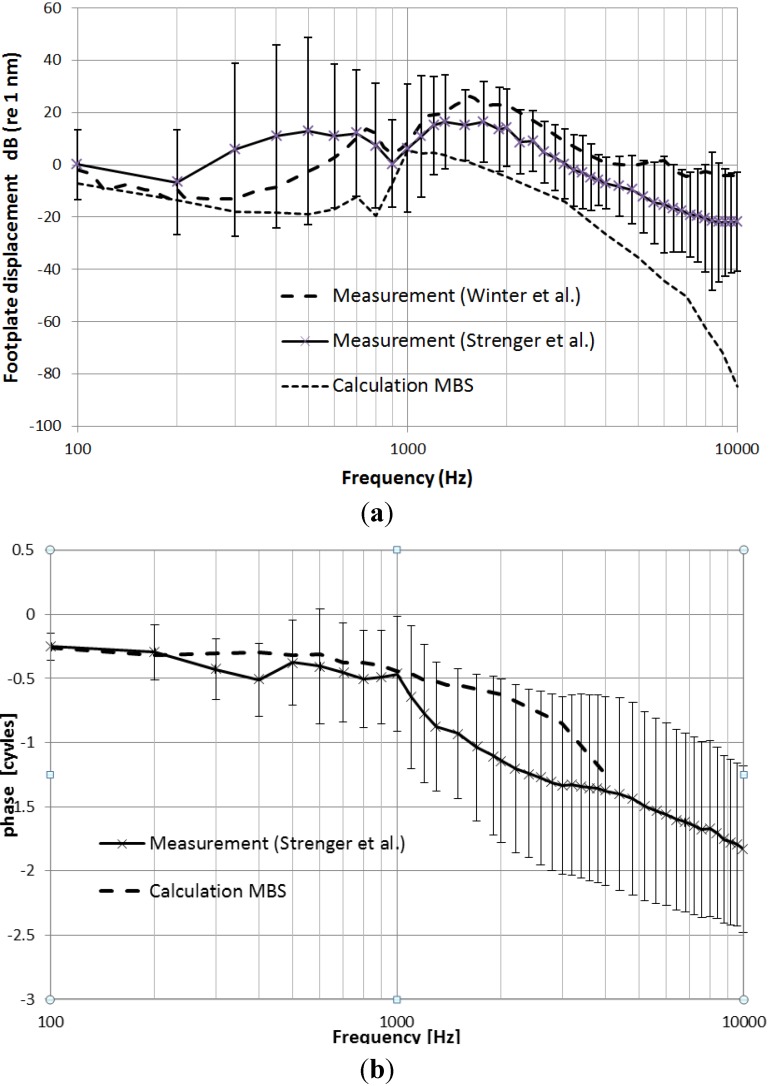
(**a**) Stapes footplate displacement as a function of frequency with FMT as the actor; calculated (MBS) displacement (dashed fine), measurement (Strenger *et al.* [18]) and measurement (Reprinted with permission from [18], Copyright 2013 German Medical Science); (**b**) Stapes footplate phase as a function of frequency with the FMT actor; calculated (MBS) displacement (dashed line) and measurement (Strenger *et al.* [18]).

The decrease of values (fine dashed) in Figure 8a displays the calculated amplitudes of SFD applying a force amplitude of 30 μN to the center of the longitudinal axis of the samarium-cobalt magnet.

The maximum displacement calculated by the MBS method is 6 dB (ref 1 nm), according to 2 nm at *f* = 1 kHz. For the frequency *f* = 800 Hz, a characteristic notch occurs. For *f* = 10 kHz, the SFD diminishes to less than −80 dB (ref 1 nm), corresponding to very low displacements of less than 0.1 pm. For comparison with the experimental results, the averaged SFDs (*n* = 5) are shown (solid line) [18]. The maximum value for the averaged displacement is 16.51 dB (ref 1 nm) or 6.69 nm at *f* = 1300 Hz, with an equivalent voltage of 10 mV supplied to the FMT to produce a maximum force amplitude of 30 μN [17]. A similar notch of approximately 10 dB, as in the computed case, is found with an upward 100 Hz frequency shift to *f* = 900 Hz in the measured case (Figure 8a). Two standard deviations are marked as error bars in both directions (higher and lower values refer to the average values). A further comparison is afforded by including the measurement result of Winter *et al.* [19] (course dashed line). The measured SFD (*n* = 1) shows a similar course, including a notch at *f* = 900 Hz. For adaption, the SFD values determined by Winter *et al.* [19] were divided by a factor of five to consider 0 mV applied to the FMT in this case, which is five-times the value used by Strenger *et al.* [18], namely, 10 mV.

### 3.2. Calculated and Measured Stapes Footplate Phases (SFP)

Figure 8b shows the calculated phase (MBS, dashed line) and the average (*n* = 5) measured phase behavior [17] for frequencies between 100 Hz and 10 kHz, including standard deviations marked as error bars. Because of the low displacement amplitudes for stimulation frequencies above 2 kHz, the standard deviations of the measured phases are considerably high (up to ±0.75 cycles). According to the stiffness dominated system at low frequencies, the shift of SFP relative to the applied voltage at the FMT is −0.25 cycles in the case of calculation and measurement. The measured phase shift decays to a value of −1.38 cycles at 4 kHz, which is on the order of the computational result. The maximum value of measured phase shift is −1.83 cycles at *f* = 10 kHz.

## 4. Discussion

We compared measured and calculated (MBS) stapes footplate displacements and phases with crimped and driven floating mass transducers (FMT), which were coupled to the long process of the incus. According to the FMT transmission factor, a peak voltage of 10 mV was applied for the resulting force amplitude of 30 μN. In the case of the MBS simulation, a force with a 30 μN amplitude value of a sinusoidal excitation was applied axially to the longitudinal axis of the FMT. The results (Figure 8a,b) show amplitudes and phases of the MBS simulation. The maximum displacement of 1.9 nm occurs at a frequency *f* = 1 kHz with decreasing amplitudes to a minimum value of 0.063 pm at *f* = 10 kHz. The phase shifts from −0.25 cycles to −1.25 cycles at 4 kHz. Because of limited numerical precision, the calculation of the phase could only be evaluated up to a maximum frequency of 4 kHz. For higher frequencies, the computational results were zero, due to the numerical imprecision resulting from the algorithm, Sodarst 2 (SIMPACK), which is an implicit formulation with automatic step size calculation. Though SFD amplitudes were calculated up to 10 kHz, the decrease to a value of −80 dB (ref 1 nm), which is 60 dB below the measured value, is arguable, because this correlates to a displacement of the stapes footplate of less than 1 zm (zeptometer, 10^−21^ m).

Figure 8a,b shows the average of five measurements of stapes footplate displacements and phases, whose individual values are given by Strenger *et al.* [18]. The maximum amplitude of the measured stapes footplate displacement is 6.69 nm (Figure 8a). A similar notch of approximately 6 dB, as it is present in the calculation at a frequency of 800 Hz, is found at 900 Hz within the average measurement. The average value of the measured phase (Figure 8b) starts at *f* = 100 Hz with zero cycles and reaches −1.18 cycles at 4 kHz (−1.25 cycles calculated by the MBS method). The computed (MBS) maximum displacement (1.9 nm) is on the order of the measured average maximum displacement (6.69 nm) with a difference of 10.93 dB. The low computed maximum value (1.9 nm) is attributed to losses in the incudostapedial joint, because the stiffness and damping of the annular ligament and the stiffness representing the cochlea impedance are comparatively low with respect to the measured cochlear input impedance of 21.1 GΩ (mks) between 0.1 and 5.0 kHz [20]. However, if a higher spring stiffness representing the cochlea input impedance was chosen, even lower amplitudes of stapes footplate displacements occur. Therefore, the calculated stapes footplate response is a reasonable compromise to adapt absolute amplitudes and frequency response by the fitting of parameters. Especially the characteristic notch shifts in dependence of the cochlea input impedance. The chosen parameters led to a notch frequency of *f* = 800 Hz. In the case of the individual temporal bone measurements, the notch frequencies varied between 800 and 1000 Hz, with a mean value of 900 Hz [17], as shown in Figure 8a. The phase courses are quantitatively comparable up to a frequency of *f* = 4 kHz. The measured and calculated (MBS) results of SFD are compared with experimental results measured by Winter *et al.* [19].

The measurement of the stapes footplate displacement with the LDV shows a similar characteristic along frequencies, though its maximum value of displacement at a frequency *f* = 1400 Hz is larger, reaching a maximum value of 20 nm, which is barely inside the positive limit of two standard deviations of the measured case. This is partly (3.01 dB) due to the differences in specifying the root mean square (RMS) in the case of the measurements of Strenger *et al.* [18] *versus* peak values in the case of Winter *et al.* [19] and, notably, because of dissimilarities in the preparations of the temporal bones. There is a high concurrence with respect to the low and high frequency characteristic of the computed and measured results presented in this work (Figure 8a,b), including the notch at 900 Hz. The slight increase of displacements at *f* = 6 kHz in the measurements of Winter *et al.* [19] might be due to a “clip” resonance, *i.e.*, an incomplete crimped fastening clip of the FMT at the long process of the incus, as it could also be seen in the measurements of Strenger *et al.* [18].

## 5. Conclusions

The segmentation and reconstruction of the middle ear components, including the FMT, affords the numerical simulation of the transfer characteristic of a middle ear electrically driven by an FMT crimped at the long process of the incus. The output force of an FMT was derived on physical principles for a well-founded stimulation of the MBS middle ear system and comparison with experimental results. The output parameter (the stapes footplate displacement amplitude and phase) could be evaluated by the MBS method and is compared with experimental results determined by laser-Doppler vibrometry. According to the age and differences in preparations of the temporal bones, the measured results have frequency-dependent standard deviations in the case of Strenger *et al.* [18]. In the case of Winter *et al.* [19], a single measurement was presented. The maximum SFD is 5.56 dB (ref 1 nm) (1.89 nm) in the case of the MBS method, 16.5 dB (ref 1 nm) (6.69 nm) in the measured case (LDV, Strenger *et al.* [18]) and 26 dB (ref 1 nm) (20 nm) in the case of Winter *et al.* [19] using LDV. Though the calculated and measured cases differ up to 31.69 dB (*f* = 500 Hz), they show a comparable behavior, especially with notches at nearly identical frequencies.

Of course, there exist alternative numerical approaches to simulate the behavior of vibrating systems. For example, the frequency transfer characteristic of a semi-implantable hearing device could be determined with a less complex procedure, namely, a two-dimensional FEM approach and a simple one-dimensional network model [21]. The frequency behavior of a device that is not implanted was simulated, and the results matched with LDV measurements. However, due to the reduced dimensionality of these methods, it is not possible to study the influence of different coupling positions on elements of the middle or inner ear within the tympanic cavity.

We conclude that the MBS method is suitable for the numerical simulation of middle ear vibrations, including middle ear hearing implants, like the FMT. Further simulations are needed that include different alternative FMT crimping places for an optimum supply for hearing impaired patients.

## Abbreviations

cgs: centimeter, gram, second units
DOF: degree of freedom
FEM: finite element method
FMT: floating mass transducer
*f*: frequency
*i*: imaginary unit
*j*: number of joints (hinges)
*L*: length
LDV: laser-Doppler vibrometry
MBS: Multi Body System
ME: middle ear
mks: meter, kilogram, second units
μ_0_: 4 × π × 10^−7^ Vs/Am magnet field constant
*N*: number of DOF of joints, Newton (unit)
*n*: number of bodies, including the inertial system
μCT: micro-computer-tomography
*R*: radius
SFD: stapes footplate displacement
SFP: stapes footplate phase
TM: tympanic membrane
ω: circular frequency
